# Case series of idiopathic intracranial hypertension in three patients with immune-complex glomerulonephritis

**DOI:** 10.1186/s12883-021-02297-3

**Published:** 2021-07-13

**Authors:** Felix Fischbach, Anne Deborah Scholz-Hehn, Christian Gerloff, Monika Pötter-Nerger

**Affiliations:** Department of Neurology, University Medical Center Hamburg-Eppendorf, Hamburg, Germany

**Keywords:** Idiopathic intracranial hypertension, Pseudotumor cerebri, Immune-complex glomerulonephritis, Nephropathy, IgA nephropathy, Case report

## Abstract

**Background:**

Idiopathic intracranial hypertension (IIH) is defined by an increased cerebrospinal fluid pressure in the absence of inflammation, structural obstructions, or mass lesions. Although the underlying pathogenesis of IIH is not fully understood, associations with specific risk factors as obesity, obstruction of cerebral venous sinuses, medications, endocrine or systemic conditions and chronic kidney disease have been described. Immune-complex glomerulonephritis as IgA-nephropathy is a frequent cause of chronic kidney failure, which was reported previously in one IIH patient. To date, there is no knowledge about the variable relation of immune-complex nephritis, kidney function and the course of IIH.

**Case presentation:**

We report three cases (two females) of concurrent diagnosis of IIH and immune-complex glomerulonephritis. All patients presented with typical IIH symptoms of headache and visual disturbances. Two patients had been diagnosed with IgA-nephropathy only few weeks prior to IIH diagnosis. The third patient had been diagnosed earlier with terminal kidney failure due to a cryoglobulin glomerulonephritis.

**Conclusion:**

We propose a possible link between renal deposition of immune-complexes and increased cerebrospinal fluid pressure. Pathophysiological hypotheses and clinical implications are discussed. We recommend clinical awareness and further systematic research to obtain more information on the association of IIH and immune-complex glomerulonephritis.

## Background

Idiopathic intracranial hypertension (IIH) is a neurological disease characterized by a cerebrospinal fluid (CSF) pressure of more than 25 cmH2O in the absence of structural obstructions or lesions. Patients typically complain about headache (76–94%) and visual disturbance due to the affection of the optic (68.72%) and abducens nerve (18%). Besides these typical symptoms patients also present with pulsatile tinnitus (52–61%), back (53%), neck (42%) or radicular (19%) pain, dizziness (52%) and cognitive disturbance (20%) [11]. Clinical criteria for diagnosis of IIH are papilledema and a normal neurological examination (except sixth cranial nerve palsy) [11]. Magnetic Resonance Imaging (MRI) criteria are (i) normal cerebral parenchyma without mass, structural lesion or meningeal enhancement, (ii) optic nerve hydrops, and (iii) empty sella sign. Obstruction of cranial venous outflow is also seen often in IIH patients. Here, two causal chains are discussed. The obstruction might be a consequence of a high intracranial pressure indicating an IIH. Also, the elevated intracranial pressure might be caused by a primary obstruction of the cranial venous outflow. Reduction of the obstruction after lumbar puncture indicates IIH. In the lumbar puncture, normal CSF constituents and an elevated CSF pressure are required [11]. Although it is occasionally referred to as “benign intracranial hypertension”, untreated IIH can cause persistent symptoms as visual loss [12]. IIH predominantly appears in obese women of childbearing age, but cases of IIH in children or older patients are documented as well [10, 14]. Despite its first description more than hundred years ago, pathogenesis of IIH still remains unclear. A variety of disorders and medications are associated with an increased prevalence of IIH. An overview is given in Table 1 derived from [11].

**Table 1:** Clinical conditions associated with intracranial hypertension derived from [11]

Haematological	Anaemia
	Polycythaemia vera
Obstruction to venous drainage	Cerebral venous sinus thrombosis
	Jugular vein thrombosis/ligation
	Superior vena cava syndrome
	Increased right heart pressure
	Arteriovenous fistulas
	Infection/subarachnoid haemorrhage causing decreased CSF absorption
Medications	Fluoroquinolones
	Tetracycline class antibiotics
	Corticosteroid withdrawal
	Danazol
	Vitamin A derivates
	Levothyroxine
	Nalidixic acid
	Tamoxifen
	Ciclosporin
	Levonorgestrel implant
	Lithium
	Growth hormone
	Indomethacin
	Cimetidine
Systemic disorders	Chronic kidney disease/renal failure
	Obstructive sleep apnoea syndrome
	Chronic obstructive pulmonary disease
	Systemic lupus erythematosus
	Psittacosis
Endocrine	Addison ‘s disease
	Adrenal insufficiency
	Cushing ‘s syndrome
	Hypoparathyroidism
	Hypothyroidism
	Hyperthyroidism
Syndromic	Down syndrome
	Craniosynostosis
	Turner syndrome

Kidney diseases have only rarely been related to IIH. Cases of two children have been published with IIH and renal transplantation [16]. Also, one case of IIH in a child receiving peritoneal dialysis has been reported [2]. One case report documented a patient with an unspecified kidney failure and IIH whose headache became less severe after starting hemodialysis [6].

Immune-complex glomerulonephritis is the most prevalent primary glomerular disorder globally, mostly IgA mediated. Most patients with renal immune-complexes remain symptom-free with an isolated microhaematuria, but mesangial deposition of immune-complexes leads to renal dysfunction in 20 – 30% of all patients [15]. There are reliable clinical predictors for renal outcome in immune-complex glomerulonephritis such as proteinuria, hypertension, and decreased estimated glomerular filtration rate (eGFR) at the time of the diagnosis [4]. To improve patient’s outcome by affecting the clinical predictors, such as hypertension, early diagnosis is required. Prompt screening for proteinuria seems to be a helpful diagnostic tool in this context.

Only one case of an adult with IIH and comorbid immune-complex-glomerulonephritis has been published [1]. Here, we report three cases of patients with immune-complex glomerulonephritis and coincident diagnosis of IIH and discuss clinical relevance and implications.

## Case presentation

### Case 1

Patient 1, a 23-year-old Caucasian overweight woman (Body-mass-index BMI = 33.8) presented with binocular blurred vision and headache, which worsened in supine position. She reported an aggravation of her visual impairment in relation to changes of light brightness. The patient reported feeling as if her vision was covered by a grey haze. This patient reported mitigation of the headache by ibuprofen intake.

A routine blood sample a few weeks before showed raised serum creatinine of 1.6 mg/dl. Urinalysis was positive for proteinuria and microhaematuria. Renal biopsy revealed an mesangioproliferative glomerulonephritis with IgA-complexes. Renal failure was classified as ‘Kidney Disease: Improving Global Outcomes’ (KDIGO) stage G3aA3. No further causes or comorbidities leading to immune-complex glomerulonephritis could be detected. At the time of clinical manifestation of arterial hypertension, a medication with ramipril was started and in the further course increased up to 10 mg/d. Albuminuria and blood pressure subsequently declined.

**Fig. 1:**
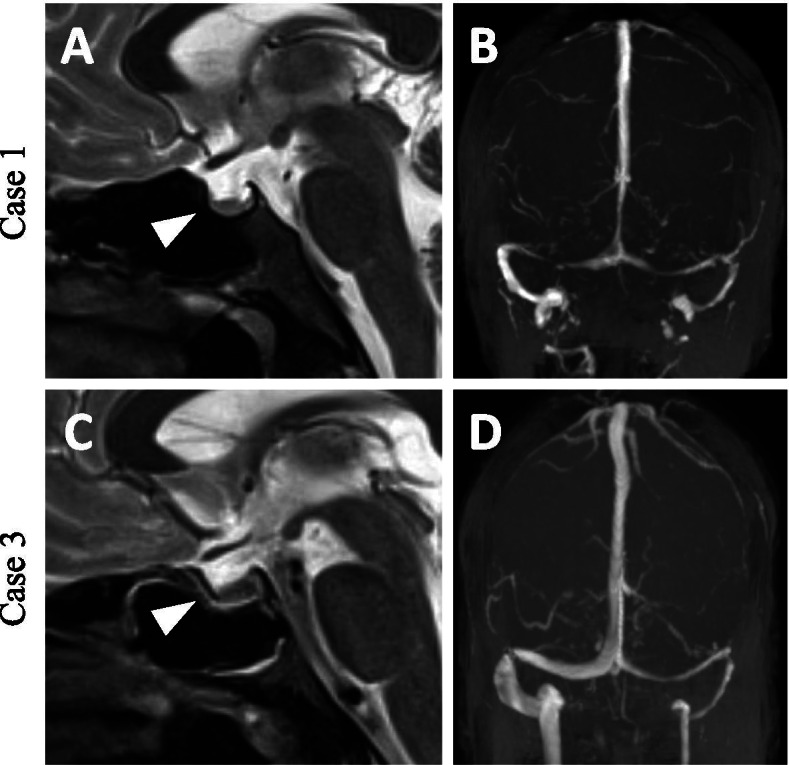
MRI scans of case 1 and 3 with typical signs of IIH. **A + C** Small pituitary gland also described as empty sella (white arrow heads). **B + D** Thin transverse sinus as an indirect sign of IIH. Note: Case 2 brought external realised MRI scan found unremarkable

Neurological clinical examination was normal, except for bilateral papilledema. MRI showed indirect signs of elevated CSF pressure (small pituitary gland). No other signs for a structural reason for elevated CSF pressure (Fig. 1) have been observed. In an initial lumbar puncture CSF opening pressure was 41 cmH2O with an unremarkable CSF composition. Besides obesity I° the patient did not show any other IIH risk factors. Following the first lumbar puncture, the patient developed a low CSF pressure syndrome (CSF pressure 6 cm H2O). After remission of the post-puncture syndrome, repeated lumbar punctures were administered and a medication with topiramate was started. The patient lost 15 kg of bodyweight (BMI = 28). In the following lumbar punctures, CSF pressure ranged from high to high-normal values (27 to 21 cm H2O) and topiramate dose could be reduced. Headache intensity and the associated ibuprofen intake declined. Serum creatinine only slightly increased to 1.7 mg/dl during the following eight months.

### Case 2

A 35-year-old African woman (BMI = 26.6) complained about headache and visual disturbance during an inpatient treatment in the department of nephrology. She had previously been diagnosed with end-stage nephropathy (KDIGO stage G5A3) and was on a medication of torsemide, ramipril and atorvastatin. At the onset of the headache, serum creatinine had peaked at 18 mg/dl. Initial diagnosis of an immune-complex-glomerulonephritis with severe diffuse tubular damage and cicatrisation was made bioptically four weeks ago. The nephropathy was mediated by a cryoglobulinaemia and a consumption of complement factor C3. As a comorbid condition, a Sjögren’s syndrome overlapping with systemic lupus erythematosus (SLE) was found two months before bilateral papilledema detection. Antinuclear antibodies (ANA), anti-Sjögren’s-syndrome-related antigen A and B (SSA, SSB) were positive. The patient had been treated with rituximab twice and corticosteroid pulse therapy. A few years before, the patient was diagnosed with hepatitis B without chronification.

Besides binocular impaired vision, which was described as visual acuity failure associated with an impression of blurred vision, no further focal neurologic signs were present. Cranial MRI scan revealed normal results without any correlates for elevated CSF pressure. In the first lumbar puncture, CSF opening pressure was 37 cmH2O without any abnormalities in laboratory analyses. Besides overweight, medical history revealed anaemia and corticosteroid withdrawal as possible predispositions for IIH. Patient underwent haemodialysis and subsequently kidney function improved but did not normalize (serum creatinine 7 – 9 mg/dl). Acetazolamide was started but was withheld only two weeks later due to side effects. Nine months later, patient presented with a BMI of 26 and without headache or impaired vision. Serum creatinine was 7.3 mg/dl. Lumbar puncture revealed (high) normal CSF opening pressure of 26 cmH2O, the patient was asymptomatic. Renal function worsened again a few weeks later (serum creatinine 11.65 mg/dl), but the patient remained symptom-free regarding IIH.

### Case 3

Patient 3 is a 32-year-old, Caucasian obese man (BMI = 38) who was diagnosed with IgA-nephropathy and arterial hypertension.

One month after the initial renal diagnosis, the patient developed symptoms of headache and blurred vision. Pain quality was described as “beating” and “squeezing”, the headache was localized fronto-temporal on both sides. The patient rated the headache intensity with a score of 7–8 on a numeric analogue scale ranging from 0 (no pain) to 10 (intolerable, devastating pain). Ibuprofen ameliorated his headache. Serum creatinine at symptom onset was 2 mg/dl. Urinalysis showed proteinuria and a few eumorphic erythrocytes. Renal biopsy showed atrophy of renal tubules. Immunohistochemistry showed glomerular deposit of IgA without signs of acute glomerulonephritis or interstitial nephritis. Renal failure could be classified as KDIGO stage G3A3. Genesis of immune-complex glomerulonephritis remained unclear.

**Fig. 2:**
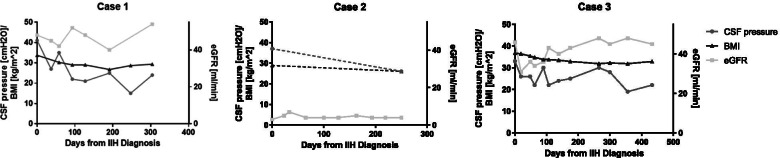
Course of clinical parameters in all reported cases: In Case 1 CSF pressure and eGFR show opposite courses by nearly stable BMI. Case 2 was lost in follow up due to treatment in another hospital. In Case 3 during time CSF pressure and BMI show a decreasing trend while eGFR increases

Cranial MRI revealed bilateral optic disc oedema and empty sella without structural causes for elevated CSF pressure (Fig. 1). The patient presented with bilateral papilledema and an initial CSF pressure of 33 cm H2O. Laboratory CSF analysis was normal. Besides obesity II° no other predispositions for IIH could be detected. Topiramate was started and monthly lumbar punctures were administered. CSF pressure ranged from 21 to 33 cmH2O. Arterial hypertension was treated with ramipril, amlodipine and hydrochlorothiazide and blood pressure normalized. Renal function slightly improved to a serum creatinine of 1.52 mg/dl during the 1.5 years of treatment. Since the initial diagnosis of IIH, the patient attained a weight loss of 10 kg (Fig. 2). Topiramate was reduced and later stopped completely. On last follow-up, the patient still negated headache or visual disturbances.

## Discussion and conclusions

Here, we report three cases of patients with diagnosed immune-complex glomerulonephritis and IIH. All patients received an extensive diagnostic workup including cranial MRI and CSF analyses. At the time of initial presentation all three patients showed an elevated BMI, which is known as a common predisposing feature for IIH [8]. Case 2 was classified as being overweight, case 1 was diagnosed with obesity I° and case 3 with obesity II°. Case 2 was also diagnosed with cryoglobulinaemia and Sjögren’s syndrome overlapping with SLE, both predispositions for developing immune-complex glomerulonephritis. Due to all these independent risk factors the observed comorbidity might be a coincidence. But in all patients IIH associated symptoms occurred in close temporal relation with the diagnosis of immune-complex glomerulonephritis, which makes a coincidental comorbidity less likely.

Pathogenesis of IIH still remains unclear. Some authors suggest a local stenosis of the transverse sinus [5], others consider the stenosis of the transverse sinus as a result of the elevated intracranial pressure. Radiological detection of asymmetric transverse sinus as seen in case 1 might be explained constitutionally as seen in a relevant percentage of the healthy population. Equally sized transverse sinuses come up only in 37–50% of all patients [7]. Since IIH mainly occurs in young obese women, hormonal, and metabolic factors such as a hypothalamic leptin-resistance, have been discussed [3]. It has also been suggested to be caused by decreased cranial venous flow due to increased intrathoracic pressure resulting from intra-abdominal obesity [9].

In literature, association of kidney function and CSF pressure has been discussed but only one case of an IIH with comorbid immune-complex IgA glomerulonephritis has been published [1]. In this special case, the immune-complex glomerulonephritis had a severe course. Our data show some association even in mild courses of immune-complex glomerulonephritis. Diagnosis of immune-complex glomerulonephritis and symptom-onset of IIH were closely related in time in all three cases. IIH diagnosis always came up second. This might be a hint towards a pathophysiological link.

The nature of this link remains speculative. Possible hypotheses might be the reduction of eGFR and consecutive hypervolemia. This fluid overload leads to elevated central venous pressure and subsequent backlog also into cranial veins. This pathophysiological condition might lead to impairment of the cranial venous outflow and finally to intracranial hypertension by malabsorption of CSF [13]. This hypothesis is in line with the mentioned theory of decreased cranial venous flow due to increased intrathoracic pressure resulting from intra-abdominal obesity [9]. Contrary to this theory, the course of eGFR and CSF pressure in Fig. 2 does not show a linear coherence. Worsening of renal function in case 2 without any signs of rising CSF pressure or clinical symptoms of IIH does not support this theory as well. The exact interaction of immune-complex glomerulonephritis and IIH remains theoretically and needs further investigations if these findings can be reproduced in different centers.

Besides the potential pathophysiological link of both diseases, our findings might be taken into consideration for the clinical workup of patients presenting with both, IgA glomerulonephritis and IIH. It seems to be justified to screen IIH patients with raised serum creatinine levels in routine blood tests for immune-complex glomerulonephritis, e.g., by searching for proteinuria or microhaematuria. A prognostic key factor is hypertension and associated proteinuria [4]. Early diagnosis of immune-complex glomerulonephritis might lead to early drug treatment of hypertension and an improved renal outcome. Also, therapeutic options of IIH in patients with reduced eGFR are limited. Both generally used drugs, topiramate and acetazolamide, must be used carefully and maybe dose reduction is necessary. Its important do detect kidney damage in IIH patients for optimising therapy.

Vice versa IIH should be considered in patients diagnosed with immune-complex glomerulonephritis if they presented with therapy-refractory headache. As shown in the medical history of our reported patients, the intake of nonsteroidal anti-inflammatory drugs (NSAIDs), such as ibuprofen, is common in IIH due to headache. Prolonged NSAID consumption may adversely affect kidney function even in the general population [17]. Early diagnosis of IIH patients with therapy-refractory headache and appropriate therapy for immune-complex glomerulonephritis might prevent prolonged NSAID consumption and renal side effects. This knowledge is indicatory for therapeutic strategies and drug recommendations given by all involved physicians.

We reported a temporal coincidence of IIH and immune-complex glomerulonephritis with different severity of mild to severe renal failure in three patients. Our findings come with therapeutic consequences as a reduction of NSAID prescription to treat IIH associated headache or a dose reduction of acetazolamide or topiramate due to reduced eGFR. Also, diagnostic screenings for a possibly comorbidity in patients with risk factors might be sensible. We suggest considering this possible association in patients presenting with both, IIH or immune-complex glomerulonephritis.

## Data Availability

All datasets on which the conclusions of the manuscript rely are presented in the main paper.

## Abbreviations

ANA: Antinuclear antibody
BMI: Body-mass-index
CSF: Cerebro spinal fluid
eGFR: Estimated glomerular filtration rate
IIH: Idiopathic intracranial hypertension
KDIGO: ‘Kidney Disease: Improving Global Outcomes’
MRI: Magnetic resonance imaging
NSAID: Nonsteroidal antiinflammatory drugs
SLE: Systemic lupus erythematosus
SSA: Anti-Sjögren’s-syndrome-related antigen A
SSB: Anti-Sjögren’s-syndrome-related antigen B
